# Supplementary material for: Jnk1 and downstream signalling hubs regulate anxiety-like behaviours in a zebrafish larvae phenotypic screen
Source: Sci Rep. 2024 May 15;14:11174. doi: 10.1038/s41598-024-61337-3 (PMC11096340; doi:10.1038/s41598-024-61337-3)
Supplement: Supplementary file 1 — Supplementary Figures. [file 41598_2024_61337_MOESM1_ESM.pdf]

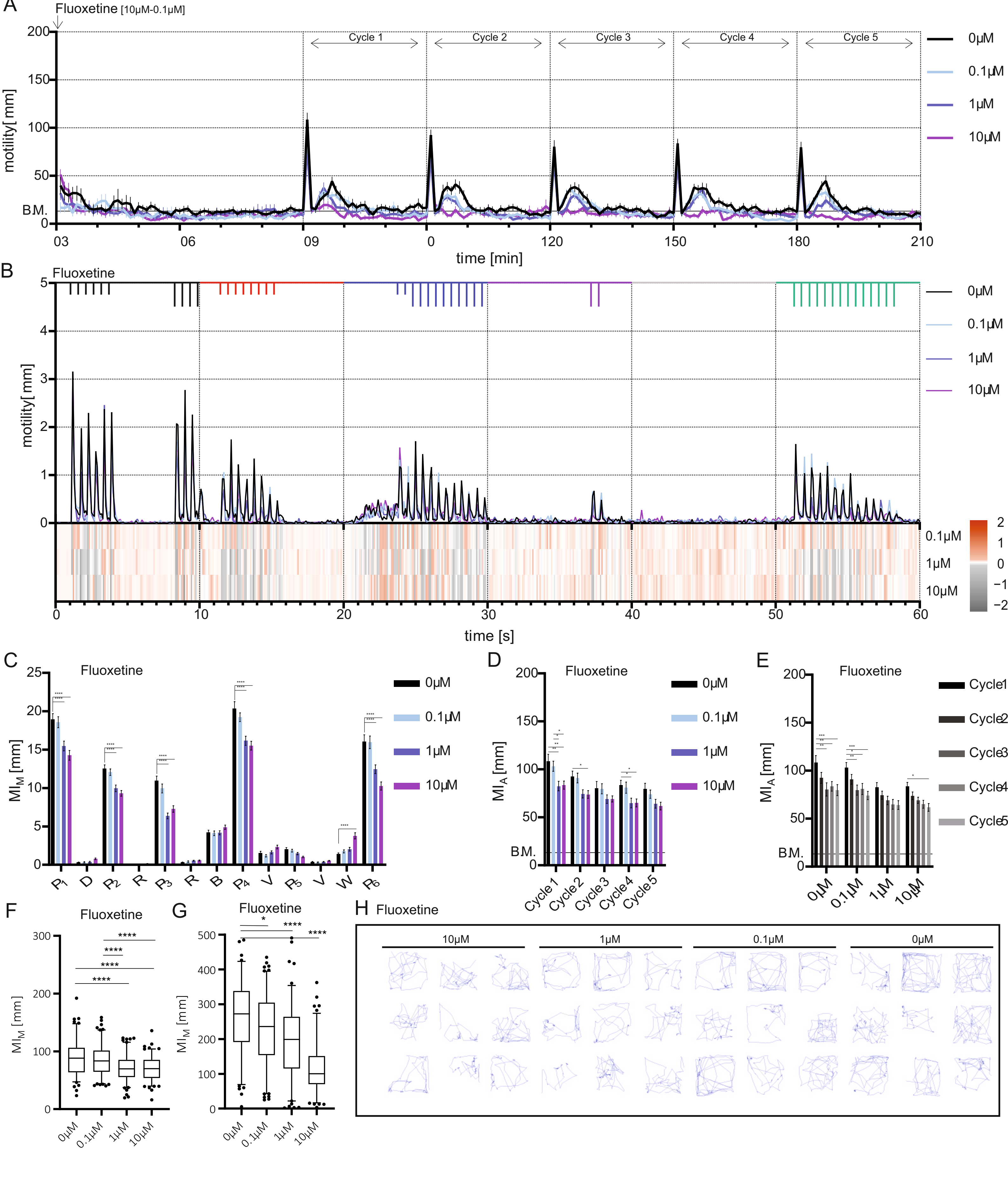

Supplementary figure 1 - Fluoxetine

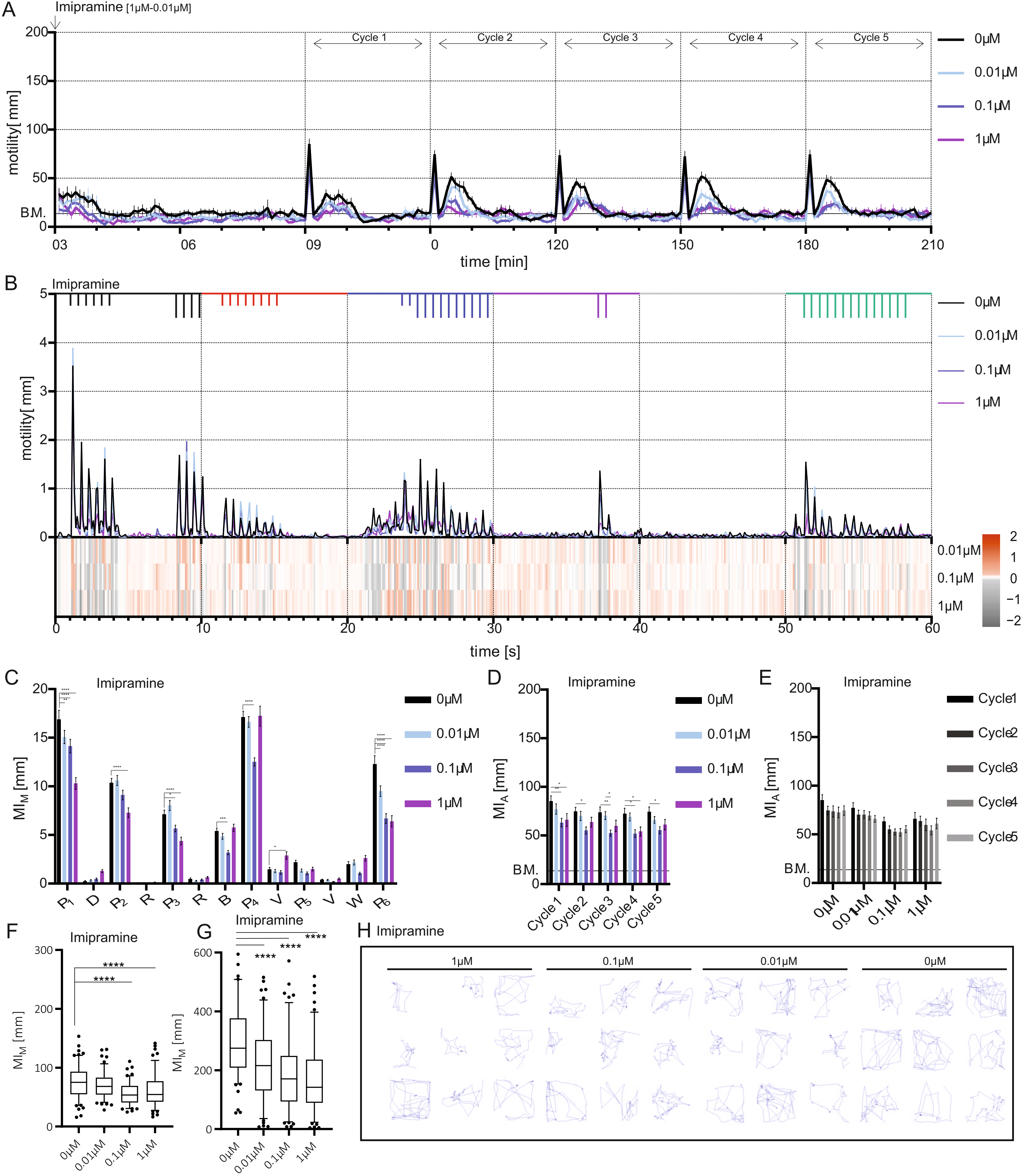

Supplementary figure 2 - Imipramine

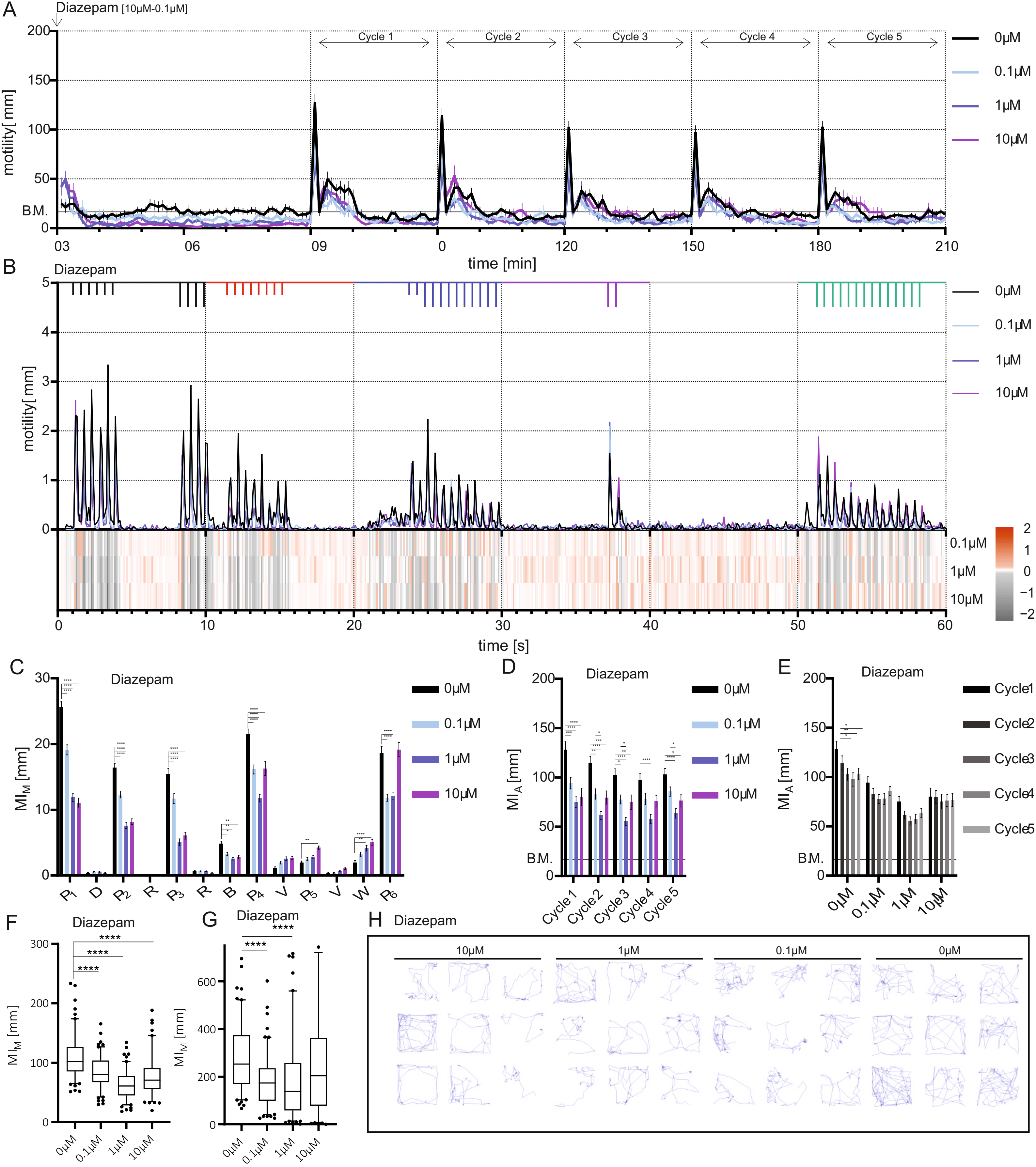

Supplementary figure 3 - Diazepam

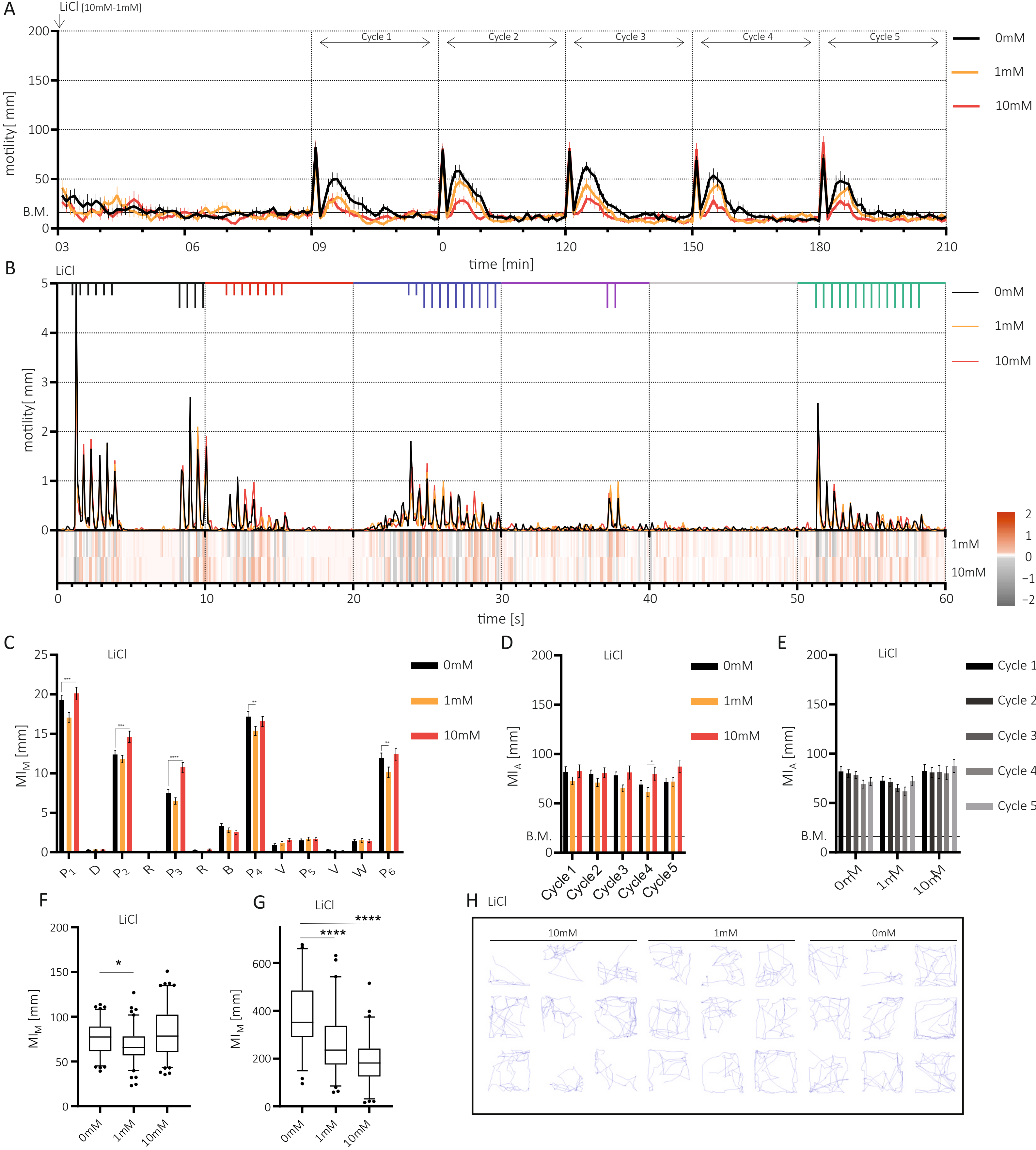

Supplementary figure 4 - Lithium

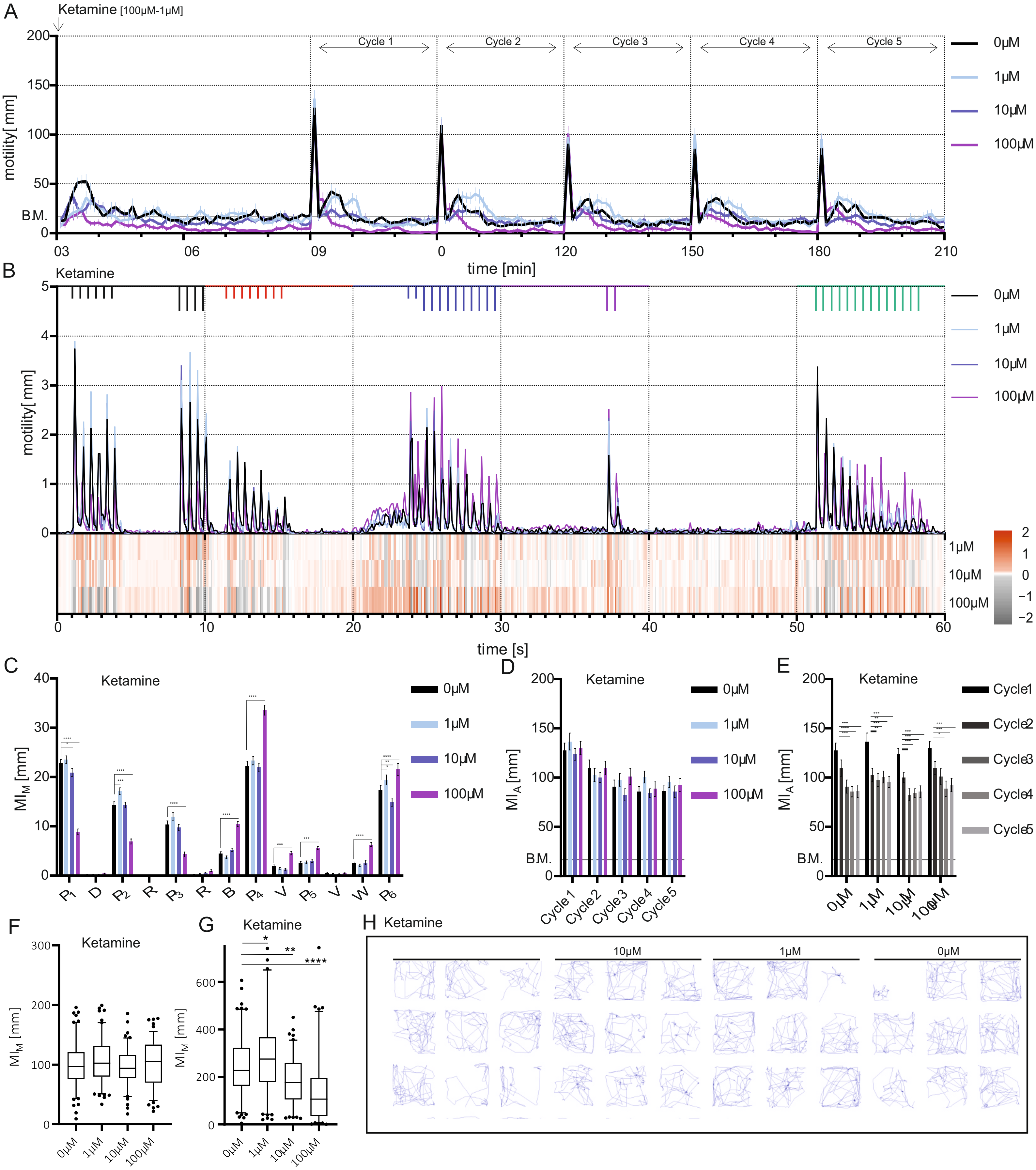

Supplementary figure 5 - Ketamine

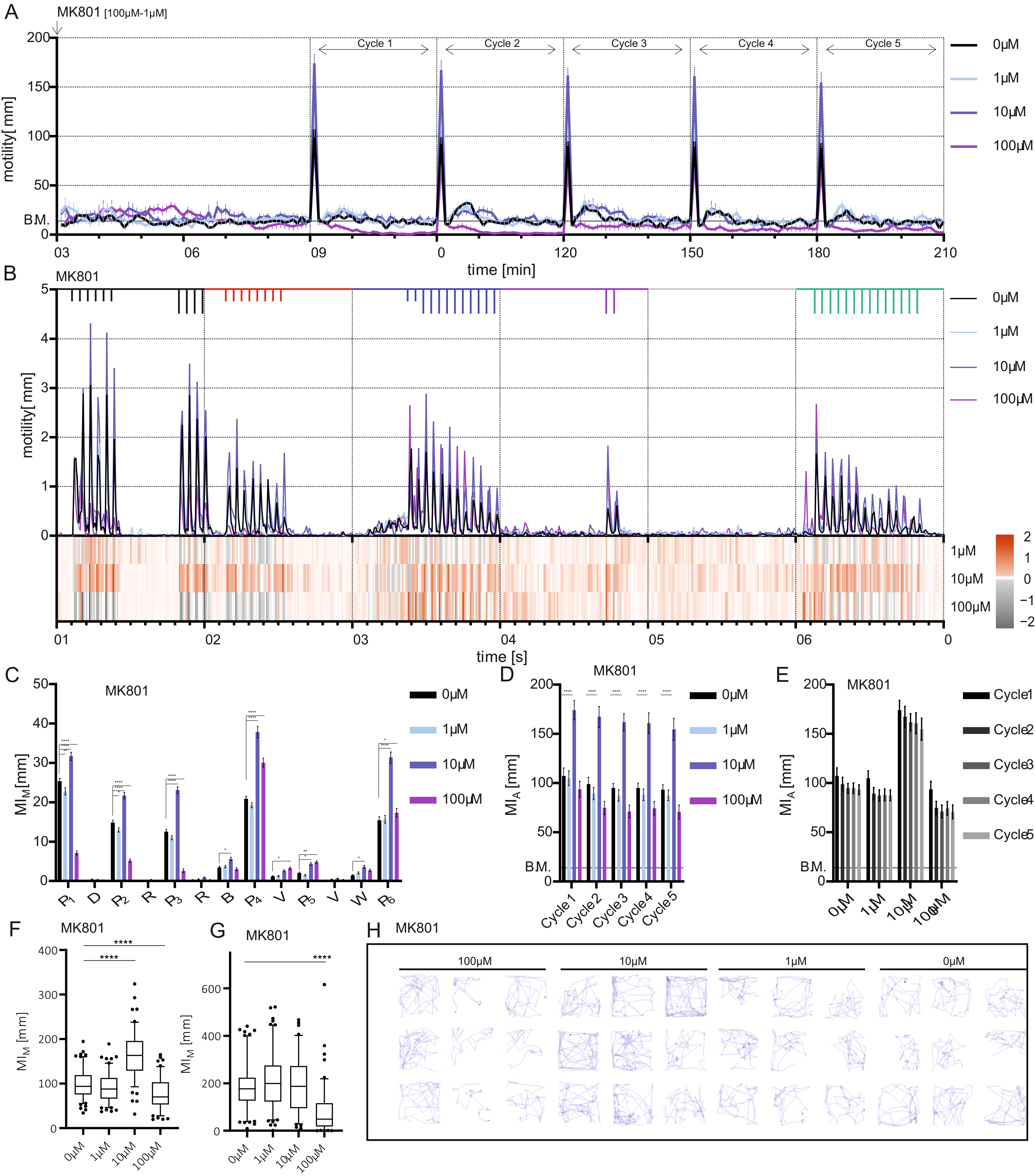

Supplementary figure 6 - MK801

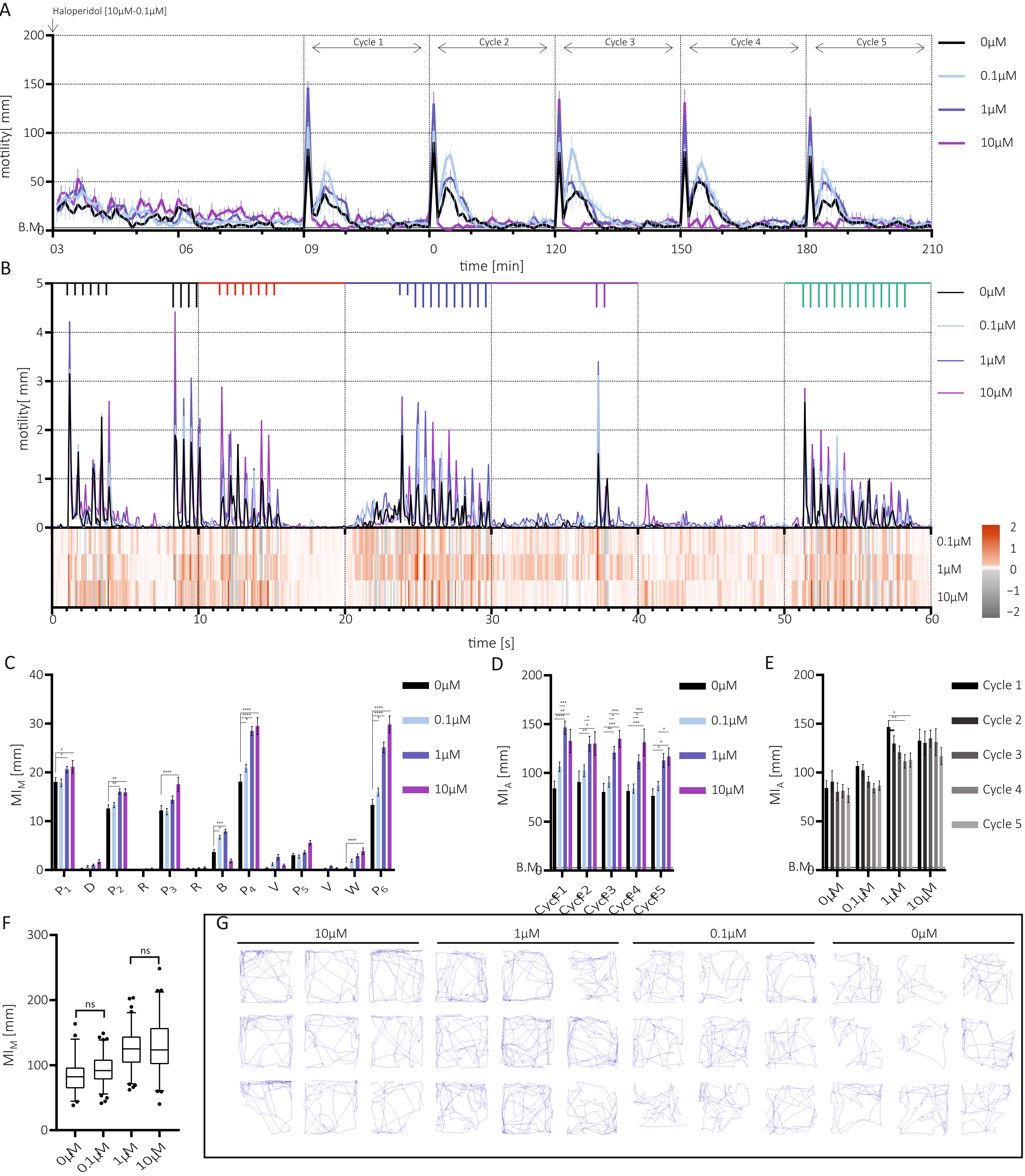

Supplementary figure 7 - Haloperidol

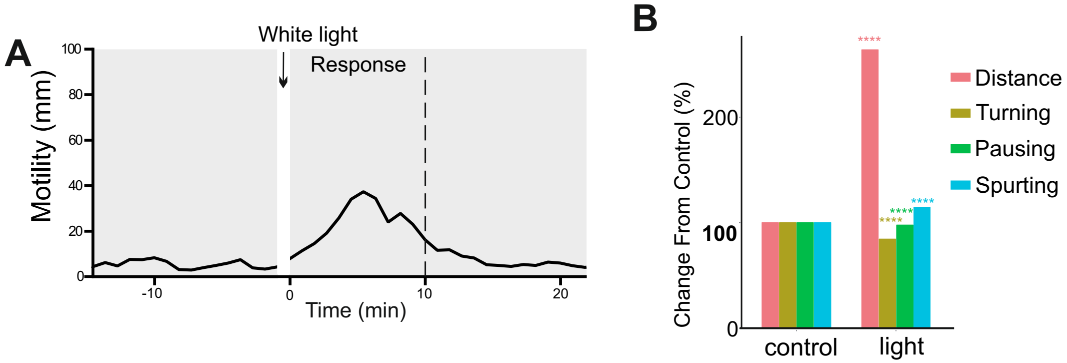

Supplementary figue 8

Supplementary Figures 1 – 7. Additional breakdown of AA drug data for fluoxetine (supplementary figure 1), imipramine (supplementary figure 2), diazepam (supplementary figure 3), lithium chloride (supplementary figure 4), ketamine (supplementary figure 5), MK801 (supplementary figure 6) and haloperidol (supplementary figure 7) are shown. For each figure, the following figure parts apply: A**.** The motility profile of zebra fish during a dose response with drug of interest. The entire test battery including 5 cycles of 1 min startle and intermission phases are shown for each drug (fluoxetine, imipramine, diazepam, LiCl and ketamine). **B.** The 1 min startle is shown for the same drugs from cycle 1 only in 0.1 s time bins. The stimuli are marked as follows: dark-red-blue-violet-white varicoloured flickering respectively. **C.** The average motility responses during the tapping (P) and light (dark, red, blue, violet, white) only stimuli as indicated P1, D, P2, R, P3, R, B, P4, V, P5, V, W, P6. **D.** Absolute motility indices (MI_A) in individual cycles. P-values from 2-way ANOVA are shown. **E.** The same data as in D plotted according to drug dosage. **F.** Drug dose response showing overall motility during the startle period. p-values were obtained using one-way ANOVA. **G.** The same as F for the post-startle response. P-values were obtained using one-way ANOVA. **H.** Representative tracking of zebrafish during cycle 1 of the 1 min startle response period. * p-value≤0.05; ** p-value *≤ 0.01; ***p-value≤0.001; ****p-value≤0.0001

Supplementary Figure 8. Behavior profiles in response to white light stressor. (A) The mean motility of zebrafish larvae following white light stress is shown for 38 larvae (5 dpf) before and after white light stress (10,000 Lux for 50 s). (B) Mean data is shown for distance, turning, pausing and spurting measured from the first 10 min following white light stress expressed as % of control response before stress. The number of fish measurements per group were: bright light = 68, control = 68. P-valueswere calculated from the original feature value distributions utilizing Wilcoxon rank sum test and adjusted with Benjamini-Hochberg procedure. * p-value≤0.05; ** p-value *≤ 0.01; ***p-value≤0.001; ****p-value≤0.0001.
